# Active Experiencing Training Improves Episodic Memory Recall in Older Adults

**DOI:** 10.3389/fnagi.2017.00133

**Published:** 2017-05-09

**Authors:** Sarah E. Banducci, Ana M. Daugherty, John R. Biggan, Gillian E. Cooke, Michelle Voss, Tony Noice, Helga Noice, Arthur F. Kramer

**Affiliations:** ^1^Beckman Institute for Advanced Science and Technology, University of Illinois at Urbana-ChampaignUrbana, IL, USA; ^2^Department of Psychology, University of Illinois at Urbana-ChampaignUrbana, IL, USA; ^3^Psychological and Brain Sciences, University of IowaIowa City, IA, USA; ^4^Department of Theatre, Elmhurst CollegeElmhurst, IL, USA; ^5^Psychology Department, Elmhurst CollegeElmhurst, IL, USA; ^6^Departments of Psychology and Mechanical and Industrial Engineering, Northeastern UniversityBoston, MA, USA

**Keywords:** aging, cognition, memory, intervention, acting, theater

## Abstract

Active experiencing (AE) is an intervention aimed at attenuating cognitive declines with mindfulness training via an immersive acting program, and has produced promising results in older adults with limited formal education. Yet, the cognitive mechanism(s) of intervention benefits and generalizability of gains across cognitive domains in the course of healthy aging is unclear. We addressed these issues in an intervention trial of older adults (*N* = 179; mean age = 69.46 years at enrollment; mean education = 16.80 years) assigned to an AE condition (*n* = 86) or an active control group (i.e., theatre history; *n* = 93) for 4 weeks. A cognitive battery was administered before and after intervention, and again at a 4-month follow-up. Group differences in change in cognition were tested in latent change score models (LCSM). In the total sample, several cognitive abilities demonstrated significant repeated-testing gains. AE produced greater gains relative to the active control only in episodic recall, with gains still evident up to 4 months after intervention. Intervention conditions were similar in the magnitude of gains in working memory, executive function and processing speed. Episodic memory is vulnerable to declines in aging and related neurodegenerative disease, and AE may be an alternative or supplement to traditional cognitive interventions with older adults.

## Introduction

Age-related cognitive declines are associated with the development of dementia (National Institutes of Health, 2011) and impact the ability of older adults to live safely and independently (Blake et al., 1988; Grundstrom et al., 2012; Boelens et al., 2013). The rate and magnitude of decline differs across cognitive functions; whereas crystalized intelligence is relatively stable, for example, episodic memory follows a steep negative trajectory (Horn and Donaldson, 1980; Lindenberger et al., 1993; Park, 2000; Bherer et al., 2013). Further, a multitude of factors appear to shape an individual’s aging trajectory (Raz and Kennedy, 2009), suggesting the possibility of intervening upon the process to potentially slow decline and promote successful aging. With this aim, various behavioral interventions have been proposed such as physical activity (Colcombe and Kramer, 2003) and cognitive training (Jaeggi et al., 2008; Karbach and Kray, 2009).

AE as a form of mindfulness training has been recently identified as a promising avenue for short-term intervention that produces lasting gains in cognitive function among older adults (Noice et al., 2004; Noice and Noice, 2009). These studies provide a good initial framework for examining AE, though, the evidence of gains is limited; the use of small samples that included individuals with elevated risk factors for cognitive decline and limited assessment of multiple cognitive abilities make the generalizability of this finding across cognitive functions in healthy aging unclear. Here, we report the largest study to date of this promising intervention in healthy older adults, including an extensive test battery of cognitive tests.

AE is a mindfulness exercise that encourages the individual to be cognizant of the immediate environment and internal state, and is commonly used as preparation for acting (Noice et al., 2004). AE was designed based on interactions with professional actors (Noice, 1996; Noice and Noice, 1999, 2001; Noice et al., 2000). The AE intervention for cognitive aging was developed by extrapolating acting strategies, including motor cues and mnemonic devices (Noice, 1996; Noice and Noice, 1999, 2006), which are often not explicitly demanded of actors but can contribute to successful script performance. AE interventions use a two-stage process of preliminary examination of the script, and engaging the character in rehearsal and performance (Noice et al., 2004). In an AE intervention, older adults with no prior acting experience are instructed to become their character and work to achieve the character’s goals (Noice et al., 2004). They do this by “actively experiencing” the character on cognitive, emotional, and physiological levels.

Previous evidence suggests that this activity may promote memory functions. Following a 4-week intervention, older adults participating in AE performed better than a no-contact control group on standardized tests of episodic memory and working memory span, but only problem solving ability was significantly improved relative to an active control that completed a visual arts course (Noice et al., 2004). Intriguingly, the AE intervention group maintained higher performance levels in these domains, with potential continued gains in episodic memory (Noice et al., 2004; Noice and Noice, 2009). Thus, AE may be an example of a brief, inexpensive, and enjoyable intervention that can have a sustainable impact on cognitive functions that typically decline during aging. Yet, in part due to the limited scope of cognitive batteries used in these prior reports, the potential mechanism(s) and specificity of AE benefits to memory function over other cognitive abilities are unclear. Unlike cognitive training regimens, AE interventions do not train participants on any specific cognitive tasks or provide any pertinent strategies that are specific to assessment.

Instead, older adults indirectly memorize a script by actively engaging in their character and in response to their acting partner (Noice et al., 2015). The intent of this exercise is to use the dialog to achieve the character’s motivation, before continuing to the next line of dialog. For example, if the script indicates a character flatter another character, then the first actor attempts to *sincerely flatter* the second actor using the exact wording in the script. Naturally, with repetition, this type of rehearsal leads to memorization of a short script (e.g., one-three pages), although it is not the explicit goal. In this manner, the AE intervention is a type of mindfulness exercise with the goal of verbatim memorization and recall of complex information, but deliberate memorization independent of the mindfulness exercise is discouraged.

Based upon the intervention design, there are two plausible routes of cognitive gains: one, promoting executive control function that is expected to have more general benefits to cognition; another, bolstering mnemonic encoding and recall that would produce more specific gains in memory and problem solving ability. For example, AE as a form of mindfulness may be similar to meditation that is hypothesized to improve attentional control in executive function (Tang et al., 2015) to confer gains in memory and reasoning abilities (Zeidan et al., 2010; Tang et al., 2012), much like the prior reports of AE. However, unlike mindfulness meditation, participants in AE have an explicit task to engage and sincerely act out a script during every rehearsal. A second hypothesized mechanism is specific to memory function. The evaluation of a character’s motivation based upon the written script and subsequent performance, and the requirement to respond in character to a dynamic scene, can be conceived as forms of memory training that encourage working memory function and episodic encoding and retrieval. The AE intervention does not explicitly train mnemonic devices, but it is plausible that the acting exercise itself promotes the use and practice of associative memory strategies that aid performance on laboratory memory tasks in older adults (Shing et al., 2008). Yet, for the lack of comprehensive assessment in previous reports, the mechanism(s) and specificity of benefits from AE to cognitive ability in older age is uncertain.

We examined these hypothesized cognitive mechanisms of AE benefits by testing intervention-related changes in several cognitive functions—executive function, episodic and working memory, and processing speed—as well as the interaction between changes in different cognitive domains. These aims are aided by a sizable sample of older adults that is the largest study of AE to date. A substantial portion of the extant evidence has come exclusively from samples drawn from the same geographic region, of older age, low-middle socioeconomic status, some living in government-subsidized retirement communities, and on average achieving a high school level education (Noice et al., 2004; Noice and Noice, 2009). Each of these factors can impact cognitive function (Jefferson et al., 2011) and it is logical that carriage of greater risk may produce larger intervention effects. The present study addresses this limitation by implementing a 4-week, randomized control trial of AE intervention among community-dwelling older adults who, on average, obtained a university degree. This sample of older adults is also double the group size of those used in previous AE studies, providing greater power to detect effects and broader representation of the general population. Further, previous reports employed an ANCOVA approach to test intervention gains, which cannot evaluate individual differences in change or the relationship between concurrent and future changes in cognition (see McArdle, 2009). Here, we use latent change models for intent-to-treat analyses, which is the gold standard (McArdle, 2009) to test changes in cognition following intervention and up to 4 months later. Within this framework, we expect the AE group to experience greater gains in cognitive function as compared to the active control group. Additionally, evidence of correlated gains in executive function, working memory and episodic memory may lend insight into the underlying mechanism of AE.

## Materials and Methods

### Participants

**Figure 1:**
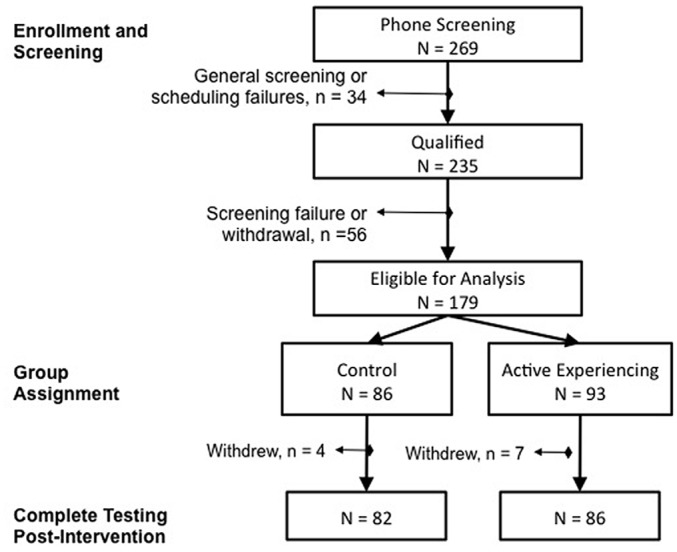
**CONSORT Flow diagram of participant recruitment, enrollment and attrition over the course of the intervention trial**.

The participants in this study were 179 community-dwelling adults aged 60–89 years (*M* = 69.46, SD = 6.59; 62% female), who on average had a college education (*M* = 16.80 years, SD = 3.48). For study enrollment, participants were right-handed, scored at least 23 on the MMSE (*M* = 28.69, SD = 1.39; Folstein et al., 1975), had no contraindication to MRI (not reported in the present article), and provided written consent for study participation. This study was carried out in accordance with the recommendations of the Institutional Review Board at the University of Illinois at Urbana-Champaign with written informed consent from all subjects. All subjects gave written informed consent in accordance with the Declaration of Helsinki. The protocol was approved by the Institutional Review Board. Based on enrollment criteria and initial phone screening, 235 persons were enrolled in the study: 179 completed the intervention and were included in analysis; another 56 individuals were removed from the study due to violations of enrollment criteria that were identified retrospectively (Figure 1). Upon successful entry into the study, participants were assigned to either the active control (*n* = 86) or the AE condition (*n* = 93). Group assignment was pseudo random depending on the participant’s time of enrollment. Attendance was monitored and participants were required to attend 75% of class sessions. The two groups were similar in age (*t*_(176)_ = −0.79, *p* = 0.43), MMSE (*t*_(176)_ = 0.22, *p* = 0.82) and years of education (*t*_(176)_ = 0.86, *p* = 0.39), as well as representation of sex ($\chi_{\left(1\right)}^{2}$ ≤ 0.15, *p* ≥ 0.70). After completing the 4-week intervention, participants returned for a post-intervention assessment (delay from the first assessment *M* = 51.48 days, SD = 14.78) and a follow-up 4 months later (delay from post-intervention assessment *M* = 127.89 days, SD = 10.35). The AE condition experienced a longer delay at post-intervention than the control (*t*_(169)_ = −3.53, *p* < 0.001) but groups were similar in follow-up delay (*t*_(143)_ = 1.78, *p* = 0.08) and delays between assessments were included as covariates in the model to account for this. Of the 179 individuals included in analyses, 33 did not return for the follow-up assessment, but were similar in demographic characteristics (all *t* < 1.55, *p* > 0.12) as those who did return and attrition was similar between the conditions ($\chi_{\left(1\right)}^{2}$ = 0.76, *p* = 0.38). Thus, intent-to-treat analyses were conducted on the total sample and missing data were handled via full information maximum likelihood (FIML) estimation with the assumption of missing at random—a non-imputation approach that leverages all available data during latent model estimation (Muthén and Khoo, 1998).

### Intervention

Participants were assigned to one of two groups: the Active Control that attended an Understanding the Art of Acting class or the intervention AE class. The control and intervention sessions occurred over a period of 4 weeks with both groups meeting two times a week for 75-min sessions, including a 15-min coffee break to encourage additional social interaction among the participants. Researchers with experience administering AE interventions chose the content of the classes and trained all outside instructors (TN and HN).

The Active Control was designed as a theater appreciation class including talks, demonstrations and video clips of stage and film performances. The course topics included the styles of acting, in addition to the history of theater. The Active Control condition was designed to rule out the possibility that learning about a popular art form like acting, along with the social interaction of being engaged in a class, are sufficient to produce the significant improvement in cognitive functioning observed in the previous theatre interventions.

The active experience training in the AE group was designed to be internally rewarding and non-competitive (Noice and Noice, 2009). The intervention has been described in detail in previous reports (Noice and Noice, 2009; Noice et al., 2015). Participants performed short scenes with a partner and scripts were 1–3 pages (large print) in length. The activities conducted in the class integrated four key concepts to teach the AE construct. First, participants were discouraged from “pretending” during sessions and instead instructed to perform every action as if it were real life. Second, participants were required to use their imaginations to mentally create the scenario in which they are asked to act. Third, participants were taught to be goal-driven by the scripted scenario and work through difficulties to achieve the character’s goal. During the first 2 weeks of AE classes, participants were not required to memorize the scripts but were encouraged to spontaneously pursue the script, by responding to interactions or alterations immediately and naturally. During the training, participants were encouraged to access each of these concepts cognitively, emotionally, and physically. These main constructs were implemented in a range of lessons throughout the intervention period. Finally, during the last 2 weeks of class, participants were expected to perform their assigned scene verbatim from memory. All participants were in the same room during classes, and the training directors circulated to provide active feedback to the acting partners. In previous studies, participants learned very short scenes in less than 1 h and longer scenes in two or three 1-h sessions (Noice and Noice, 2009; Noice et al., 2015).

### Cognitive Measures

Tasks were selected to address a range of cognitive domains including episodic memory, working memory, semantic knowledge, executive function and processing speed. All computer-based tasks were designed in E-prime version 1.1 (Psychology Software Tools, Pittsburgh, PA, USA) and administered on computers with 17 inch cathode ray tube (CRT) monitors. The same tests were repeated at pre- and post-intervention, and at the follow-up after 4 months. All assessments were administered and scored by research assistants who were blinded to group assignment.

#### Episodic Memory

The Logical Memory task was taken from the Virginia Cognitive Aging Project (Salthouse and Ferrer-Caja, 2003; Salthouse, 2004, 2005, 2010). Two recorded stories were played for the participant. Each story contained 25 specific story details and eight thematic details. Immediately following the story, participants were asked to recall the story in as much detail as possible. Following the second story, participants were asked to recall as much detail as possible from the first story without having it played back, providing a measure of delayed recall. Number of details recalled for the specific story and thematic categories were used as separate measures of episodic memory. Latent constructs for story and thematic recall were each defined by immediate and delayed recall.

#### Spatial Working Memory

The computerized spatial working memory (SWM) task was administered at variable difficulty memory loads of two, three and four target dots, randomly arranged on the computer screen. Participants completed 12 practice trials followed by 120 test trials. The dots were visible for 500 ms or 1000 ms, replaced with a fixation cross for 3000 ms. Following the fixation, a red dot either appeared in the same location where one of the black dots was or in an altered location. Participants indicated whether the dots were in the original or altered positions. Accuracy of responses for each of the three conditions was used as measures to identify the latent construct.

#### Verbal Working Memory

In the computerized N-back test, participants viewed a series of letters presented sequentially for 500 ms with an inter-trial interval of 2000 ms. Participants performed both a 1-back and a 2-back condition, with five runs of 20 letters presented for each condition. In the 1-back condition, participants were instructed to respond by pressing a button when the currently presented letter was the same as the previously presented letter (match trial), but to press a different button when the current letter did not match the previously presented letter. Instructions were similar for the 2-back condition that required participants to indicate if the currently presented letter was the same or different to the letter presented 2 trials previously. For both 1- and 2-back conditions, 50% of trials met the match rule. Response accuracy on each the 1- and 2-back conditions were used as measures to identify the latent construct.

#### Semantic Memory

Category fluency of animals and fruits/vegetables was administered to measure semantic memory. Participants were prompted with a category and orally recalled as many items as possible in that category within 1 min. The total number of correct responses per category was used as indicators of category fluency.

#### Executive Function

The computerized Task Switching test consisted of two individual tasks, each presenting a single digit (1–9, excluding 5) for 2500 ms. Participants were required to make a judgment about the presented digit based upon the background color. When presented on a pink background, participants were instructed to indicate if the digit was more or less than 5. When presented on a blue background, participants indicated if the digit was odd or even. Participants completed one block of 40 trials of each task rule individually followed by a block of 160 trials that required switching between response rules, designed to present randomly. The latent construct was defined by accuracy on the switch block and average accuracy of the non-switch blocks.

#### Processing Speed

The latent processing speed construct was defined by two measures: Part A of the Trail Making Test and Digit Symbol Substitution Task. In Part A of the Trail Making Test, the participant was required to connect a total of 25 numbers (1–25) in ascending and alphabetical order, as quickly as possible without removing the pencil from the page. Longer completion time indicates slower processing speed. In the Digit Symbol Substitution Task participants were provided nine pairs of numbers and symbols, and then were required to fill in the matching symbol for a provided number using the key as a reference. There were 133 possible items and participants were given 2 min to complete as many items as possible working from left to right without leaving any blank. Greater number of correct responses was an index of faster processing speed.

### Statistical Analyses

All analyses were completed in a latent modeling framework, estimated in MPlus software (v. 7; Muthen and Muthen). Changes in cognitive ability from pre- to post-intervention, and from post-intervention to follow-up after 4 months, were estimated in latent change score models (LCSM). A LCSM is similar to a difference score, but because it is determined by latent constructs, estimates of change and individual differences therein are free of measurement error (McArdle, 2009). In this model construction, sequential changes between measurement occasions were correlated, and pre-intervention performance was allowed to correlate with subsequent change.

To construct the LCSM, several measurement features were imposed. Latent constructs were each determined by multiple measures, fixing one measure factor loading to 1, the other loadings were freely estimated, and all measurement residuals were freely estimated. The following latent constructs were estimated (*italics indicates factor loading fixed to* 1 f*or latent identification*): story and thematic recall were each defined by *immediate* and delayed recall; category fluency by accuracy of recall for *animal* and vegetable/fruit categories; SWM by accuracy on the *one*, two and three dot conditions; verbal working memory by accuracy on *2-back* and 1-back; task switching by accuracy on the *switch* and non-switch blocks; and processing speed by performance on *digit symbol* and Trails Part A (see Table 1). To ensure measurement invariance longitudinally, several constraints were added to the LCSM: estimated factor loadings were constrained to be equal over time, measure intercepts and variances were equal at each time point, and repeated measures were allowed to correlate but the magnitude of the correlation was constrained to be equal between occasions. There were a few exceptions: measurement variance of the 3-dot condition of the SWM task was freely estimated and measure intercepts of the task switching block were freely estimated.

**Table 1:** **Mean latent change at post-intervention and the 4-month follow-up in the total sample**.

		Post-intervention			4 Month follow-up		
Construct	Pre-intervention variance	Mean change [BS 95% CI]	Variance in change	*d*	Mean change [BS 95% CI]	Variance in change	*d*
Story recall	0.73*	0.32* [0.21/0.42]	0.49*	0.37	0.12 [0.02/0.25]	0.50*	0.14
Thematic recall	0.73*	0.18^†^ [0.04/0.32]	0.87*	0.21	0.12 [0.01/0.26]	0.60*	0.14
Category fluency	0.61*	0.07 [−0.10/0.23]	1.00*	0.09	0.21* [0.09/0.36]	0.59*	0.27
Spatial WM	0.89*	−0.04 [−0.20/0.12]	1.04*	−0.04	0.12 [−0.02/0.26]	1.00*	0.13
Verbal WM	0.25*	0.09 [−0.03/0.19]	0.27*	0.18	0.06 [−0.01/0.17]	0.17*	0.12
Task switching	1.00*	0.14^†^ [0.02/0.26]	0.70*	0.14	0.16^†^ [0.03/0.30]	0.77*	0.16
Processing speed	0.85*	0.21* [0.13/0.28]	0.07	0.23	0.07 [−0.01/0.14]	0.08	0.08

Note: Unstandardized change scores reported for measures normed to the mean and standard deviation of the total sample at pre-intervention. All constructs are latent composites of multiple measures.*p < 0.01, ^†^p < 0.05, α’ = 0.01. d is a standardized coefficient of change: d = Mean Change/√(Pre-intervention Variance). BS 95% CI, bias-corrected bootstrapped 95% confidence intervals; WM, working memory.

Prior to model construction, all measures were normed to pre-intervention scores in the total sample. Thus, change scores can be interpreted as standardized change from pre-intervention. All reported effects are unstandardized coefficients. A standardized effect size of mean latent change was calculated: *d* = (Mean Latent Change)/√(Pre-intervention Latent Variance). Cognitive constructs that evidenced significant individual differences in change were further tested for covariates, including age, sex, delay between occasions, as well as group differences. Intervention group differences were tested in a grouped LCSM that included constraints for measurement invariance also between groups. Only the means and variances tested for group differences were freely estimated. To test whether changes in one cognitive ability predict change in another following intervention, parallel change score models were constructed. These included correlated change in cognitive constructs at each assessment occasion, as well as change from pre- to post-intervention in one cognitive ability predicting future change from post-intervention to follow-up in another ability. Model fit was determined by several accepted indices (Hu and Bentler, 1999): non-significant normal theory weighted chi-square (χ^2^), comparative fit index (CFI > 0.90), root mean square error of approximation (RMSEA < 0.05), and standardized root mean residual (SRMR < 0.08). Model fit was determined for the total sample and with grouped modeling procedures.

Analyses were completed with the assumption of intent-to-treat and included the total sample. Missing data were handled via FIML—a non-imputation approach that leverages all available data during effect estimation (Muthén and Khoo, 1998; Larsen, 2011), and the current recommended practice for longitudinal studies with attrition (Little and Card, 2013). To avoid spurious results due to a smaller sample size, all LCSM were bootstrapped with bias-correction (5000 draws; Hayes and Scharkow, 2013) to produce 95% confidence intervals (BS 95% CI) of unstandardized effects. Due to the limitation on the number of parameters that can be reasonably estimated in proportion to the sample size, each cognitive construct was evaluated in a separate model. A Bonferroni correction was made for multiple comparisons (α’ = 0.01).

## Results

### Longitudinal Consistency in Measures

Prior to testing longitudinal change, longitudinal consistency of the measures was evaluated with Pearson correlations between pre-intervention measures and subsequent testings. Performance on all tasks had acceptable longitudinal consistency: the highest consistency in performance on digit symbol (*r* = 0.85 and 0.83), and the lowest on n-back task 1-back (*r* = 0.30 and 0.59) and 2-back conditions (*r* = 0.55 and 0.49).

### Latent Longitudinal Change in Cognitive Ability

Within the entire sample, mean changes from pre- to post-intervention and from post-intervention to the follow-up 4 months later were tested in sequential LCSM. All models had excellent fit: $\chi_{\left(7–31\right)}^{2}$ = 8.73–40.49, *p* = 0.05–0.79; CFI = 0.97–1.00; RMSEA = 0.00–0.07; SRMR = 0.02–0.05. Story recall improved at post-intervention (mean = 0.32, *p* < 0.001, α’ = 0.01; BS 95% CI: 0.21/0.42), as did thematic recall (mean = 0.18, *p* = 0.03, α’ = 0.01; BS 95% CI: 0.04/0.32), although the effect did not survive correction for multiple comparisons. Both tasks evidenced additional gains at follow-up 4 months later, supported by BS 95% CI that do not overlap with zero, but neither effect reached statistical significance (Table 2). Task switching also showed nominal gains at post-intervention (mean = 0.14, *p* = 0.04, α’ = 0.01) and follow-up (mean = 0.16, *p* = 0.04, α’ = 0.01), whereas category fluency only improved at follow-up (0.21, *p* = 0.005, α’ = 0.01; BS 95% CI: 0.09/0.36). Performance on all other tasks was stable over the course of study (Table 2). However, individuals varied in the magnitude of gains in performance on all constructs, except processing speed, and we went on to test possible intervention group differences in performance changes. In addition to intervention group differences, several covariates were tested to explain individual differences in change: pre-intervention performance, age, sex and the delay between assessments as control variables, as well as correlated changes at post-intervention and follow-up. See Table 2 for a summary of all covariates to change. Better performance at pre-intervention was associated with lesser gain at post-intervention in all constructs (*r* = −0.48 to −0.10, *p* < 0.001); the same pattern was not consistently observed at follow-up, but individuals who showed greater gains at post-intervention experienced lesser gain at follow-up (*r* = −0.34 to −0.23, *p* < 0.001). Final models of covariates had good fit: $\chi_{\left(28–55\right)}^{2}$ = 31.11–59.45, *p* = 0.01–0.33; CFI > 0.95; RMSEA < 0.06; SRMR < 0.06. Possible intervention effects were tested as group differences in the magnitude of change in each cognitive domain, accounting for pre-intervention performance, age and delay between assessments as covariates.

**Table 2:** **Covariates of latent change**.

Construct	Change score	Pre-intervention performance	Age	Sex	Delay
Story recall	Post-intervention	−0.23*	−0.08	0.18	−0.02
	Follow-up	−0.10	0.14^†^	−0.03	−0.08
Thematic recall	Post-intervention	−0.59*	0.06	0.23	−0.10
	Follow-up	−0.20	−0.01	−0.24	0.01
Category fluency	Post-intervention	−0.56*	0.33*	−0.08	0.00
	Follow-up	0.44*	−0.39*	0.21	0.01
Spatial WM	Post-intervention	−0.30*	−0.05	0.08	−0.06
	Follow-up	0.06	−0.10	0.00	−0.05
Verbal WM	Post-intervention	−0.10*	−0.11^†^	0.12	0.00
	Follow-up	0.01	0.10^†^	−0.13	0.02
Task switching	Post-intervention	−0.33*	−0.04	0.22	−0.08
	Follow-up	−0.24*	0.07	−0.16	−0.01

Note: All covariate effects estimated in latent change score models. Unstandardized coefficients reported for measures normed to pre-intervention means and standard deviations in the total sample. *p < 0.01,^†^p < 0.05, α’ = 0.01. Constructs are latent composites of multiple measures. WM, working memory.

### Intervention Group Differences

Prior to evaluating group differences in latent change, groups were confirmed to be statistically similar in performance on cognitive measures at pre-intervention (all *t*_(176)_ = −1.28 to 1.90, *p* > 0.06), except the AE condition performed worse on immediate story recall (*t*_(176)_ = 2.09, *p* < 0.04) and immediate (*t*_(176)_ = 2.39, *p* = 0.02) and delayed (*t*_(176)_ = 3.18, *p* = 0.002) thematic recall. In latent models testing group differences in change, we constrained pre-intervention latent episodic memory scores to be equal between groups to confirm that this was not a bias in the analysis.

**Figure 2:**
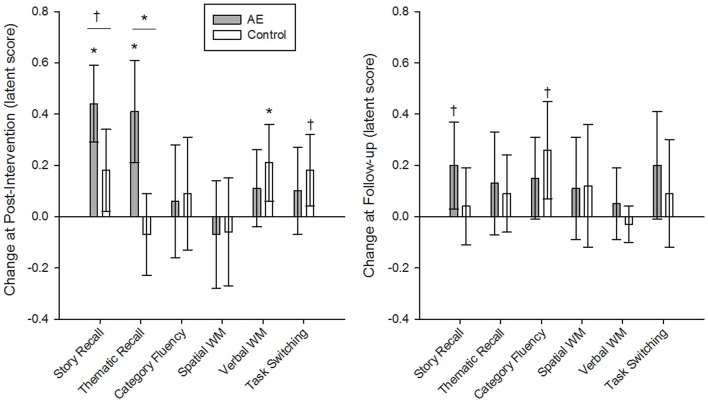
**Intervention group differences in latent change in cognitive ability at post-intervention and at follow-up, 4 months later**. The Active experiencing (AE) condition produced significantly greater gains in story and thematic recall in episodic memory as compared to the control group at post-intervention, but not at follow-up. Groups were similar in the magnitude of change in all other cognitive constructs. Intervention group comparisons were made in a grouped latent change score model, including covariates and constraints to ensure measurement invariance longitudinally and between groups. Unstandardized coefficients are reported for measures normed to pre-intervention means and standard deviations of the total sample. Error bars represent bias-corrected bootstrapped 95% confidence intervals; *p < 0.01; ^†^*p* < 0.05****; α’ = 0.01. AE, AE intervention condition; Control, active control condition.

The AE intervention produced greater gains in episodic memory than Active Control. Grouped models of episodic memory had excellent fit (story and thematic recall, respectively): $\chi_{\left(62\right)}^{2}$ = 78.51 and 65.78, *p* = 0.08 and 0.35 (AE = 39.49 and 43.23; Active Control = 39.02 and 22.55); CFI = 0.97 and 0.99; RMSEA = 0.06 and 0.03; SRMR = 0.06. The AE condition experienced significant improvement in story recall at post-intervention (0.44, *p* < 0.001; BS 95% CI: 0.31/0.59) and a trend for the same at follow-up (0.20, *p* = 0.05, BS 95% CI: 0.04/0.37) whereas the Active Control group showed no significant change in performance over the study (Table 3). Indeed, the intervention produced significantly greater gains at post-intervention as compared to Active Control (difference = 0.26, *p* = 0.04, BS 95% CI: 0.07/0.46), but additional gains at follow-up were similar between groups (difference = 0.16, *p* = 0.25, BS 95% CI: −0.06/0.38; Figure 2). A similar pattern was observed for thematic recall—the AE intervention produced greater gains at post-intervention (difference = 0.49, *p* = 0.002, BS 95% CI: 0.23/0.75) but not at follow-up (difference = 0.04, *p* = 0.78, BS 95% CI: −0.19/0.29). Although the magnitude of change did not significantly differ between groups at follow-up, the intervention produced a different pattern of gains in episodic memory over the course of study—suggesting better maintenance (and potentially continued gains) of recall ability up to 4 months after the intervention. To ensure that this intervention-related effect was not an artifact of group differences in pre-intervention performance level, we imposed additional model constraints that held groups to be equal at pre-intervention, and the AE condition still demonstrated greater gains in thematic recall at post-intervention (difference = 0.40, *p* = 0.01; BS 95% CI: 0.14/0.66) and the test of the effect in story recall (difference = 0.19, *p* = 0.10; BS 95% CI: 0.00/0.38) was likely underpowered based upon the BS 95% CI that slightly overlapped with zero. Therefore, the evidence of group differences in repeated-testing gains in episodic memory is likely an intervention effect and not a bias from a possible ceiling effect in performance.

**Table 3:** **Intervention group differences in change at post-intervention and 4-month follow-up**.

	Change at Post-Intervention			Change at Follow-up		
Construct	AE [BS 95% CI]	Control [BS 95% CI]	Difference [BS 95% CI]	AE [BS 95% CI]	Control [BS 95% CI]	Difference [BS 95% CI]
Story recall	0.44* [0.31/0.59]	0.18 [0.02/0.33]	0.26^†^ [0.07/0.46]	0.20^†^ [0.04/0.37]	0.04 [−0.10/0.19]	0.16 [−0.06/0.38]
Thematic recall	0.41* [0.22/0.64]	−0.07 [−0.21/0.09]	0.49* [0.23/0.75]	0.13 [−0.05/0.33]	0.09 [−0.06/0.24]	0.04 [−0.19/0.29]
Category fluency	0.06 [−0.15/0.28]	0.09 [−0.17/0.33]	−0.03 [−0.35/0.31]	0.15 [−0.01/0.32]	0.26^†^ [0.07/0.47]	−0.10 [−0.39/0.13]
Spatial WM	−0.07 [−0.28/0.13]	−0.06 [−0.27/0.16]	−0.01 [−0.29/0.27]	0.11 [−0.06/0.31]	0.12 [−0.12/0.36]	−0.01 [−0.31/0.29]
Verbal WM	0.11 [−0.05/0.26]	0.21* [0.07/0.36]	−0.10 [−0.32/0.09]	0.05 [−0.05/0.19]	−0.03 [−0.10/0.09]	0.07 [−0.07/0.22]
Task switching	0.10 [−0.14/0.27]	0.18^†^ [0.04/0.32]	−0.08 [−0.34/0.15]	0.20 [0.03/0.41]	0.09 [−0.10/0.32]	0.11 [−0.18/0.38]

Note: Intervention group comparisons were made in a grouped latent change score model, including covariates and constraints to ensure measurement invariance longitudinally and between groups. Unstandardized coefficients are reported for measures normed to pre-intervention means and standard deviations of the total sample.*p < 0.01; ^†^p < 0.05; α’ = 0.01. AE, active experiencing intervention condition; Control, active control condition; BS 95% CI, bias-corrected bootstrapped 95% confidence intervals; WM, working memory.

Group differences were only detected in episodic memory ability and groups were equivalent in changes in all other constructs (Table 3). All other group models of cognitive ability had excellent fit: $\chi_{\left(46–117\right)}^{2}$ = 46.65–131.46, *p* = 0.17–0.45 (AE = 25.17–54.55, Active Control = 21.48–76.91); CFI > 0.98; RMSEA < 0.04; SRMR < 0.07. Except the model of task switching that had less-than-optimal fit due to violations to the assumption of measurement invariance between groups: $\chi_{\left(55\right)}^{2}$ = 96.33, *p* < 0.001; CFI = 0.96; RMSEA = 0.09; SRMR = 0.09.

### Correlated Changes in Cognition

Although groups did not differ in the magnitude of change in any domain besides episodic memory, individuals varied in changes in several cognitive domains and the pattern of correlated changes across domains may lend insight into the mechanism of AE intervention benefits. In a parallel latent change score model, we evaluated correlations between concurrent changes in cognitive constructs, including story and thematic recall, executive function and working memory. This was first examined in the total sample. As expected, individuals who experienced greater gains in story recall also showed gains in thematic recall immediately following the intervention (*r* = 0.25, *p* < 0.001) and at follow-up (*r* = 0.19, *p* < 0.001). Greater gains in executive function from post-intervention to follow-up also correlated with concurrent gains in thematic recall (*r* = 0.11, *p* = 0.03) but not in story recall at that time (*r* = 0.07, *p* = 0.17), and changes from pre- to post-intervention were not correlated with concurrent changes in thematic (*r* = 0.001, *p* = 0.99) or story recall (*r* = 0.04, *p* = 0.44). Changes in working memory were unrelated to concurrent change in episodic recall at post-intervention (*r* = −0.06 and *r* = 0.02, *p* > 0.09, story and thematic recall, respectively) or at follow-up (*r* = 0.01 and *r* = 0.00, *p* > 0.77, respectively). Thus, while performance on story and thematic recall was correlated, it did not consistently associate with concurrent changes in executive function or working memory.

To further evaluate a possible cognitive mechanism of AE intervention gains in episodic memory, the parallel change score model included a test of change in executive function and working memory from pre- to post-intervention predicting future change in episodic recall from post-intervention to follow-up. However, there was no evidence of change in one cognitive domain predicting future change in another. Change in executive function immediately after the intervention did not predict future change in episodic recall assessed 4 months later (*b* = −0.11 and *b* = −0.06, *p* > 0.29, story and thematic recall, respectively), nor did change in working memory (*b* = −0.17 and *b* = −0.12, *p* > 0.46, story and thematic recall, respectively). Due to the lack of evidence for these effects in the total sample, intervention group differences were not further tested. Taken together, group intervention effects were limited to episodic recall and gains in this cognitive domain were unrelated to changes in executive function and working memory.

## Discussion

Typical aging is characterized by cumulative and progressive declines in cognition (Horn and Donaldson, 1980; Lindenberger et al., 1993; Park, 2000; Bherer et al., 2013) and the prospect of interventions to slow this decline is intriguing. AE, a form of mindfulness, is a promising intervention that has not been widely explored. Here, we find that a group of older, community-dwelling adults who completed a 4-week AE intervention experienced greater repeated-testing gains in episodic memory recall than the Active Control group. These gains in function were maintained up to 4 months later, although the intervention groups did not significantly differ in performance at follow-up. This is the largest study of AE to date and included a broad range of cognitive assessments. Yet, there was no evidence of intervention benefits to other cognitive abilities, suggesting that AE may specifically bolster episodic memory in healthy aging. Thus, the mechanism of the AE intervention is likely closely related to mnemonic encoding and recall to confer gains within this domain, albeit without additional global cognitive benefits.

The pace and magnitude of declines during aging vary across cognitive domains and episodic memory appears to be particularly sensitive (Horn and Donaldson, 1980; Lindenberger et al., 1993; Park, 2000; Bherer et al., 2013). Thus, short-term interventions that bolster this function with sustained benefits could have major implications for public health. Several intervention approaches, including aerobic exercise (Colcombe and Kramer, 2003; Smith et al., 2010; Roig et al., 2013), cognitive training (Melby-Lervåg and Hulme, 2013), and mindfulness meditation (Tang et al., 2015) have been tested and produce mixed results in improving memory function in older adults. Here, we partially replicate previous reports of AE improving memory function relative to active control groups (Noice et al., 2004; Noice and Noice, 2009). Importantly, the present study reports an extensive range of cognitive assessments never before administered in this type of intervention, of which only episodic memory showed improvements. This result indicates a potential selective intervention benefit via a mechanism specific to mnemonic function, and not global improvements in executive function or working memory, for example.

Although we find evidence for a selective effect, we can only speculate on the precise mechanism by which AE bolsters memory ability, and it is plausible that it relates to improved use of mnemonic strategies for encoding and retrieval. The AE training required the participant to evaluate a character’s motivation and affect in the course of performing a script. Although participants were not instructed to deliberately memorize the script, the repetition and creative development of the character in relation to the scene performance is conceptually similar to elaborative mnemonic strategies that improve subsequent recall in the laboratory (Hertzog and Dunlosky, 2004; Preston and Eichenbaum, 2013). Older adults appear to spontaneously use such strategies less frequently and less effectively than their younger counterparts do, partially explaining worse recall accuracy in later life (Kausler, 1994; Verhaeghen and Marcoen, 1994). Moreover, age-related deficits in episodic recall can be mitigated by supplying deep encoding strategies (Shing et al., 2008) that are similar to those derived from AE training. AE does not explicitly train mnemonic strategies or specific cognitive functions, yet rehearsal over 4 weeks may have encouraged older adults to spontaneously use deep encoding strategies more frequency and effectively when performing tasks other than acting. In this regard, AE training may have implicit benefits to episodic memory function. Moreover, these benefits were sustained up to 4 months later, similar to the long-term benefits of mnemonic strategy training evidenced years after intervention (Gross and Rebok, 2011). However, without independent reports of strategy use, we can only speculate on the source of gains in episodic recall.

An alternative hypothesized mechanism of AE benefits to memory and problem solving that have been reported previously (Noice et al., 2004; Noice and Noice, 2009) is a boost in executive control functions that, in turn, promotes cognitive ability, similar to the putative mechanism of benefits following mindfulness meditation (Tang et al., 2012, 2015). However, we found no evidence of intervention gains in other cognitive abilities, including executive function and working memory, and individual differences in the magnitude of change in these domains did not predict subsequent change in episodic recall. Thus, the AE intervention does not appear to directly target executive function *per se*.

The present results are not completely consistent with previous reports of AE (Noice et al., 2004; Noice and Noice, 2009). This may in part be due to differences in sample characteristics. Although the health of previously reported samples was not thoroughly documented (Noice et al., 2004; Noice and Noice, 2009), previous reports were on samples that were older, of lower SES and education level than participants in the current study. Each of these demographics are proxies for multifarious processes in aging that have been shown to predict steeper cognitive declines (Jefferson et al., 2011). Because greater risk can moderate the magnitude of intervention effects (e.g., Colcombe and Kramer, 2003; Smith et al., 2010; Danielsson et al., 2015), the generalizability of AE benefits in cognition to samples with lesser concomitant risk can be questioned. Here, we partially replicate the previous reports in a college-educated and healthy aging sample, and failure to find effects outside of episodic memory may reflect the sample selection. Future studies should consider additional health factors that may account for individual differences in responsiveness to the intervention. Moreover, individual differences in brain structure and function may interact with health factors and demonstrate change in response to the intervention to further explain cognitive function, as has been documented with mindfulness meditation (Tang et al., 2015). We aim to address these hypotheses in future reports and intentionally limited this initial report to the analysis of primary cognitive outcomes. Nonetheless, the moderate effect sizes within episodic memory function measured in this sample and the sustained effect 4 months later demonstrate the promise of the AE intervention in the course of normal cognitive aging.

The current report replicates and expands the extant literature on AE and employs a robust analytic approach. Yet, the evidence should be interpreted with consideration of several limitations. In addition to the possible bias introduced by strict sample selection from the Champaign-Urbana, IL metro area, the sample was compromised by some attrition. However, this is the largest sample to date testing the effects of AE. Further, intent-to-treat analyses were completed on the entire sample and we handled missing data via FIML—a non-imputation approach that leverages all covariance information available during model estimation. To avoid spurious results from the smaller sample size, estimated effects were bootstrapped with bias-correction to produce 95% confidence intervals. Yet, we cannot completely eliminate possible bias related to sampling characteristics. A second limitation of the study is pseudo randomization of group assignments, which may be reflected in group differences in episodic memory performance at pre-intervention. The latent models that assessed intervention group differences included constraints to account for this, but we cannot completely account for this possible source of bias. Future studies should consider a true randomization scheme. A third limitation is our assessment of sustained benefits only 4 months after intervention. Longer delays with multiple measurement occasions are necessary to evaluate this further. Despite these limitations, we offer promising evidence of AE intervention benefits to episodic memory function in healthy aging and identify propitious avenues of future study.

## Conclusion

Previous reports of AE identified it as a promising intervention to promote cognitive function into older age, yet the mechanism of intervention benefits as well as the generalizability of gains across cognitive domains in the course of healthy aging had not yet been examined. Here, we identified specific gains in episodic recall from AE relative to the Active Control, but no other evidence of intervention gains in cognition. The cognitive mechanism of AE intervention benefits appears to be specific to mnemonic encoding and retrieval, as individual differences in executive function and working memory were unrelated to subsequent change in episodic recall. Episodic memory is particularly vulnerable to decline in aging and here we find promising evidence of intervention benefits in a healthy aging sample that is larger than any previously reported in an AE intervention. Because sustainable benefits were seen after a relatively brief intervention, AE may be a promising activity to slow episodic memory declines that are typical in aging. However, despite the extensive neuropsychological battery, no other cognitive domains exhibited benefits from the AE intervention, in contrast to expectations and previous reports. Future studies that include additional measures of brain structure and function, and other health factors, may substantiate AE as an effective intervention to promote successful aging.

## Author Contributions

SEB, AMD, JRB, GEC, MV, TN, HN and AFK: manuscript preparation and editing; SEB, AMD, JRB, GEC and MV: data analysis and interpretation; GEC, MV, TN, HN and AFK: study design; AFK, TN and HN: study funding.

## Conflict of Interest Statement

The authors declare that the research was conducted in the absence of any commercial or financial relationships that could be construed as a potential conflict of interest.
